# Book Reviews

**Published:** 1875-03-15

**Authors:** 


THE DISEASES OF THE STOMACH. By Wilson Fox, M.D., etc. Philadelphia: H. C. Lea. 8vo.; 280 pp.

This, under a new title, is really a third revised and enlarged edition of the author's little treatise on the diagnosis and treatment of the varieties of dyspepsia. The present edition is much more complete and comprehensive in its scope and character, including all the diseases of the stomach.

The author's name and reputation as a writer and authority upon this subject, renders superfluous an extended comment upon the excellence of the work.

---

CLINICAL LECTURES ON DISEASES OF THE URINARY ORGANS, Delivered at University College Hospital, by Sir Henry Thompson. Philadelphia: H. C. Lea. For sale by Jansen, McClurg & Co., Chicago. 8vo; 200 pp. Price $2.25.

This is the second American, reprinted from the third London edition of this little work. The author's aim, he states, has been to produce in the smallest possible compass, an epitome of practical knowledge concerning the nature and treatment of diseases of the urinary organs. That this aim of the author has been accomplished, is sufficiently proven by the abundant success of the former editions.

---

DENTAL PATHOLOGY AND SURGERY. By S. J. A. Salter, M.D. New York: Wm. Wood & Co. 8vo; 400 pp.

This seems to be a very complete and comprehensive treatise on dental pathology and surgery. The author has devoted himself exclusively for the past twenty-three years to the practice of dental surgery, and as dental surgeon to Guy's Hospital, has had abundant field for study and experience in this special department. The work is well and numerously illustrated. Covering, as it does, a special field which is but imperfectly elaborated in the works on general surgery, the surgeon will find it a valuable and useful addition to his library.

---

A SERIES OF AMERICAN CLINICAL LECTURES, Edited by E. C. Seguin, M.D. Vol. I, No. 1. ON DISEASE OF THE HIP JOINT, by Lewis A. Sagre, M.D. New York: G. P. Putnam's Sons. For sale by Keen, Cooke & Co., Chicago. 24 pp. Price 40c.

This is the first issue of the proposed series of American Clinical Lectures. The numbers, for the present, are to be issued monthly, and sold separately, at from thirty to fifty cents each. The contributors to succeeding numbers, so far as announced, are Prof. Austin Flint, Sr., Prof. A. L. Loomis, Prof. A. Jacobi, Prof. T. G. Thomas, Prof. W. H. Thompson, Prof. H. R. Sands, and Prof. W. H. Draper, all of New York.

